# Power of computed-tomography-defined sarcopenia for prediction of morbidity after pancreaticoduodenectomy

**DOI:** 10.1186/s12880-019-0332-6

**Published:** 2019-04-27

**Authors:** Nicolas Linder, Alexander Schaudinn, Katharina Langenhan, Felix Krenzien, Hans-Michael Hau, Christian Benzing, Georgi Atanasov, Moritz Schmelzle, Thomas Kahn, Harald Busse, Michael Bartels, Ulf Neumann, Georg Wiltberger

**Affiliations:** 1 Department of Diagnostic and Interventional Radiology, University of Leipzig, Leipzig, Germany; 2 Department of Surgery, Campus Virchow and Campus Mitte, Charité – Universitätsmedizin Berlin, Berlin, Germany; 3 Department of Visceral, Transplantation, Thoracic, and Vascular Surgery, University Hospital Leipzig, Leipzig, Germany; 4 Department of General- and Visceral Surgery, Helios Clinic Leipzig, Leipzig, Germany; 5 Department of General, Visceral, and Transplantation Surgery, University Hospital of RWTH Aachen, Aachen, Germany

**Keywords:** Postoperative pancreatic fistula, Sarcopenia, Mean muscle attenuation, Fat segmentation, Computed tomography

## Abstract

**Background:**

The goal of our study was to evaluate the current approach in prediction of postoperative major complications after pancreaticoduodenectomy (PD), especially symptomatic pancreatic fistula (POPF), using parameters derived from computed tomography (CT).

**Methods:**

Patients after PD were prospectively collected in a database of the local department of surgery and all patients with CT scans available were assessed in this study. CT parameters were measured at the level of the intervertebral disc L3/L4 and consisted of the areas of the visceral adipose tissue (A_VAT_), the diameters of the pancreatic parenchyma (DPP) and the pancreatic duct (DPD), the areas of ventral abdominal wall muscle (A_MVEN_), psoas muscle (A_MPSO_), paraspinal muscle (A_MSPI_), total muscle (A_MTOT_), as well as the mean muscle attenuation (MA) and skeletal muscle index (SMI). Mann-Whitney-U Test for two independent samples and binary logistic regression were used for statistical analysis.

**Results:**

One hundred thirty-nine patients (55 females, 84 males) were included. DPD was 2.9 mm (Range 0.7–10.7) on median and more narrow in patients with complications equal to or greater stadium IIIb (*p* < 0.04) and severe POPF (*p* < 0.01). DPP median value was 17 (6.9–37.9) mm and there was no significant difference regarding major complications or POPF. A_VAT_ showed a median value of 127.5 (14.5–473.0) cm^2^ and was significantly larger in patients with POPF (*p <* 0.01), but not in cases of major complications (*p* < 0.06). A_MPSO_, A_MSPI_, A_MVEN_ and A_MTOT_ showed no significant differences between major complications and POPF. MA was both lower in groups with major complications (*p <* 0.01) and POPF (*p <* 0.01). SMI failed to differentiate between patients with or without major complications or POPF.

**Conclusion:**

Besides the known factors visceral obesity and narrowness of the pancreatic duct, the mean muscle attenuation can easily be examined on routine preoperative CT scans and seems to be promising parameter to predict postoperative complications and POPF.

## Background

Pancreaticoduodenectomy (PD) is a well-established procedure with low mortality rate in centers of high patient volume [1]. However, the morbidity rate still reaches up to 50% [2], most prominent due to postoperative pancreatic fistulas (POPF). In preparation for PD, patients receive computed tomography (CT) and derived parameters are widely available. Therefore, various efforts were undertaken to predict and stratify the risk of major complications and in particular of POPF based on preoperative CT data with partly controversial results:

The majority of recent studies evaluated the influence of visceral adipose tissue (VAT), on complications and POPF [3–6]. However, the measures were often not in accordance to the standard procedure, e.g. in regards to the anatomical site of measurement [4, 7], or dependent on expensive segmentation tools [4, 5, 8]. Both facts are limiting a more standardized and clinical use of this parameter. Results were heterogeneous, but most publications showed a positive influence of visceral adipose tissue on complications [3–5], only few showed contradicting results [7].

Another focus of previous studies was to characterized certain structures of the pancreas on CT images, such as the diameter of the pancreatic duct (DPD), the diameter of the pancreatic parenchyma (DPP), the mean density of the gland or the estimated postoperative remnant volume [4–6, 8–17]. Most of these parameters were too complex for clinical routine relying on special image reformation. Therefore only the diameters of the pancreatic parenchyma and duct seem to be reliable enough for further evaluation and showed promising results in earlier reports .

The evaluation of sarcopenic obesity using CT data was introduced in 2013 [18], but only recently this concept was also applied to predict major complications and POPF after PD [8]. Nishida et al. could show that preoperative sarcopenia is a strong and independent risk factor to predict POPF. In this publication 49.6% of the patients were classified as sarcopenic and POPF was significantly higher in these patients, however the results of a Japanese cohort can hardly be applied to a western cohort due to differences in body composition [19].

Although the results found by Nishida et al. are promising they cannot be directly transferred to a western cohort and additionally they lacked a key parameter of sarcopenic obese, the mean attenuation (MA) of skeletal muscle. MA is reported in Hounsfield units and can indicate elevated levels of intramuscular fat accumulation, which is ignored when solely quantifying the muscle area. MA seems to be a powerful prognostic tool, however its capability to predict major complication after PD was never evaluated.

The goal of our study was therefore first to provide an overview over the reported parameters for prediction of major complications and POPF, second to validate selected methods on our own patient cohort using a freely available, custom-made software tool and third to further evaluate the role of sarcopenic obesity by quantifying MA for the first time in this specific context.

## Methods

### Obtaining clinical data

A database of patients receiving pancreatic resection was prospectively established at the Department of Visceral, Transplantation, Thoracic, and Vascular Surgery, University Hospital Leipzig, Germany. For this retrospective descriptive current study, all patients with preoperative CT datasets available, not older than 2.5 months prior to surgery, were analyzed. The study was approved by the local ethics committee of the University of Leipzig (ID: 276–13-07102013) and written informed consent was waived. Authors from the Department of Surgery (FK, HH, CB, GA, MS, MB, GW) had access to the complete clinical data for patient selection. Image readers (NL and AS) had access to patient name and date of birth only.

All patients with malignant perimampullar tumors (ductal adenocarcinoma of the pancreas, distal bile duct carcinoma, duodenal carcinoma and ampullar carcinoma), premalignant neoplasm and benign tumor eg pseudotumor after chronic pancreatitis were included, Further inclusion criteria were pancreatic head resection (eg PPPD or Kausch-Whipple-procedure). All patients with multivisceral resection (liver, colon, spleen, kidney and small bowel) and vascular resection were excluded. All patients received CT-scan of the abdomen and thorax for local staging and to exclude distant metastasis. Routine blood parameter and tumor marker were obtained in each patient.

Besides CT data the patient characteristics sex, age and body mass index (BMI) were assessed: Postoperative morbidity was classified according to the Clavien-Dindo-Classification [20], POPF according to the International Study Group for Pancreatic Fistula (ISGPF) criteria [2]. Briefly the Clavien-Dindo-Classification is a simple and valid measure to assess surgical outcomes based on complications ranging from “deviations of the normal postoperative course” (grade I) to “death” (grade V) with a marked transition to more invasive procedures requiring general anesthesia at grade IIIb. The ISGPF criteria are also clear cut and routinely used. POPF grade A represents transient fistula and lacks clinical impact, POPF grade B leads to adjustments in treatment such as repositioned drainage and POPF grade C demands a major change in the clinical management such as invasive procedures like a percutaneous drainage. Additionally POPF Grade C usually extends the hospital stay. Further baseline characteristics are provided in Table 1.

**Table 1:** Baseline characteristics

	Complication < IIIb	Complication IIIb ≤	*p<*	No Grade B or C fistula	Grade B or C fistula	*p<*
Patient data						
Gender (female / male)	39 / 63	16 / 21	0.60	42 / 71	13 / 13	0.23
Age (years)	60.6	65.6	0.18	60.9	62.0	0.81
BMI (kg/m^2^)	24.9	26.2	**0.03**	24.8	26.9	**0.05**
Comorbidities						
cardiovascular	55	29	**0.01**	63	21	0.21
cerebrovascular	4	2	0.71	5	1	0.89
pulmonary	14	12	**0.01**	16	10	**0.01**
endocrine	49	18	0.99	54	13	0.87
gastrointestinal	61	21	0.70	66	16	0.80
hemato−/oncologic	7	2	0.75	8	1	0.54
Immunologic	4	2	0.71	4	2	0.36
gynecologic	12	6	0.50	15	3	0.80
urologic	15	6	0.84	16	5	0.53
chronic pancreatits	34	9	0.30	37	6	0.33
metabolic syndrome	2	3	**0.09**	4	1	0.95
arterial hypertension	48	27	**0.01**	54	21	**0.01**
obesity	11	10	0.20	14	7	0.06
new diabetes mellitus	7	1	0.35	8	0	0.16
impaired physical performance	18	7	0.83	22	3	0.37
loss of appetite	18	9	0.35	20	7	0.25
feeling of abdominal pressure	40	10	0.21	40	10	0.69
ascites	2	0	0.40	2	0	0.50
jaundice	48	17	0.98	54	11	0.70
peripheral edema	2	0	0.40	2	0	0.50
fever	7	3	0.78	7	3	0.32
night sweats	6	1	0.44	6	1	0.75

Continuous parameters are reported as median, and categorical parameters are counted. *p*: level of significance was determined by the Mann-Whitney-U Test. bold numbers indicate *p* < 0.05

### Surgical procedure and perioperative management

Surgical reconstruction was done in a standardized fashion with a retrocolic end-to-side pancreaticojejunostomy and two surgical drains in the abdomen. Somatostatin was not used routinely. Mean follow-up time for major complications and POPF included total hospital stay and 3 months after discharge. However, readmission data may be insufficient due to the fact that patients with minor post-discharged-complications might have to be submitted to other local hospitals.

Diagnosis and definition of POPF was according to the official guidelines of the International Study Group of Pancreatic Fistula (ISGPS). In detail, drain amylase was routinely measured 3 days after surgery. If amylase was negative drain was removed. If amylase was increased (>three times higher serum levels) drain was not removed. Only in patients with clinical signs of infection and/or elevated infection parameters CT-scan was performed. A routine ultrasound or CT-scan were not performed. Patients with symptomatic POPF were treated with parenteral nutrition, a systemic antibiotic therapy and interventional drainage in cases of accessible fluid collections.

### CT technique

Patients received preoperative abdominal CT scan within 75 days prior to surgery (mean 16 days). A majority of 133 of 139 CT scans was performed as standard staging CT (one single venous phase with 120 kV, weight dependent i.v. injection of Iodine of 400 mg/mL without oral contrast), most frequently in a 64 slice CT scanner (Brilliance 64, Philips Medical System, Best, Netherlands). Slice thickness was mainly 3 or 5 mm. Patients were examined in supine position.

### Analysis of radiological data

**Fig. 1:**
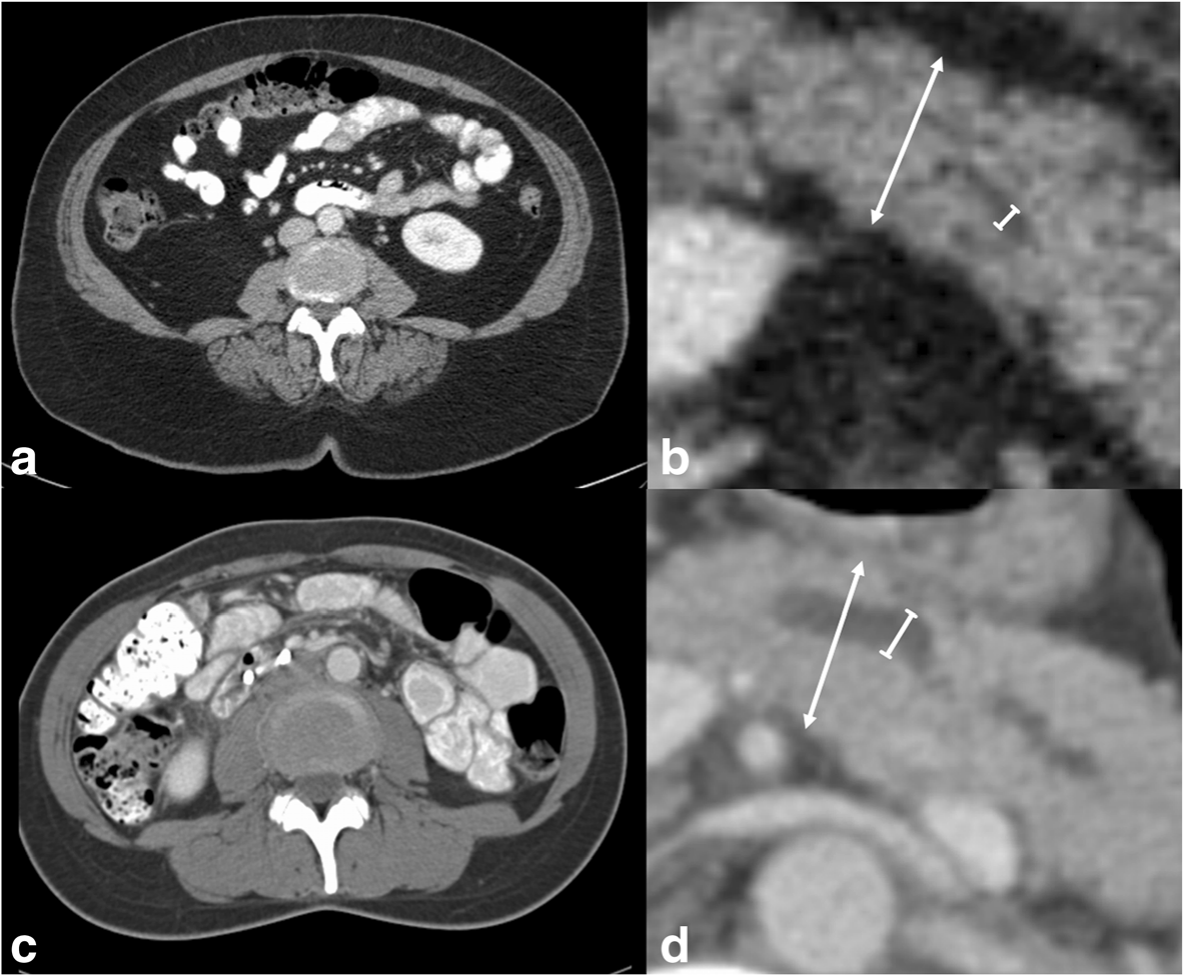
Sample CT images of two female patients with high (**a**, **b**) and low (**c**, **d**) risk profile for postoperative fistula and complications. a,b. Sample patient with grade C fistula and grade IIIb complications. A_VAT_ (**a**) was 203.2 cm^2^, DPP was 19.7 mm and DPD was 2.3 mm, respectively (arrow and line in **b**). **c**, **d**. Female patient with no fistula and no complications. A_VAT_ (**d**) was 26.6 cm^2^, DPP was 18.7 mm and DPD was 4.0 mm, respectively (arrow and line in **d**)

For measurement of the diameters of pancreatic parenchyma and pancreatic duct, a slice was individually selected showing the pancreas corpus. Measurement was carried manually using standard PACS tools (see Fig. 1) and in accordance with earlier reports [12]. For analysis of VAT, skeletal muscle areas and muscle attenuation, the axial slice at the level of the intervertebral space L3-L4 was chosen [21–26].

**Fig. 2:**
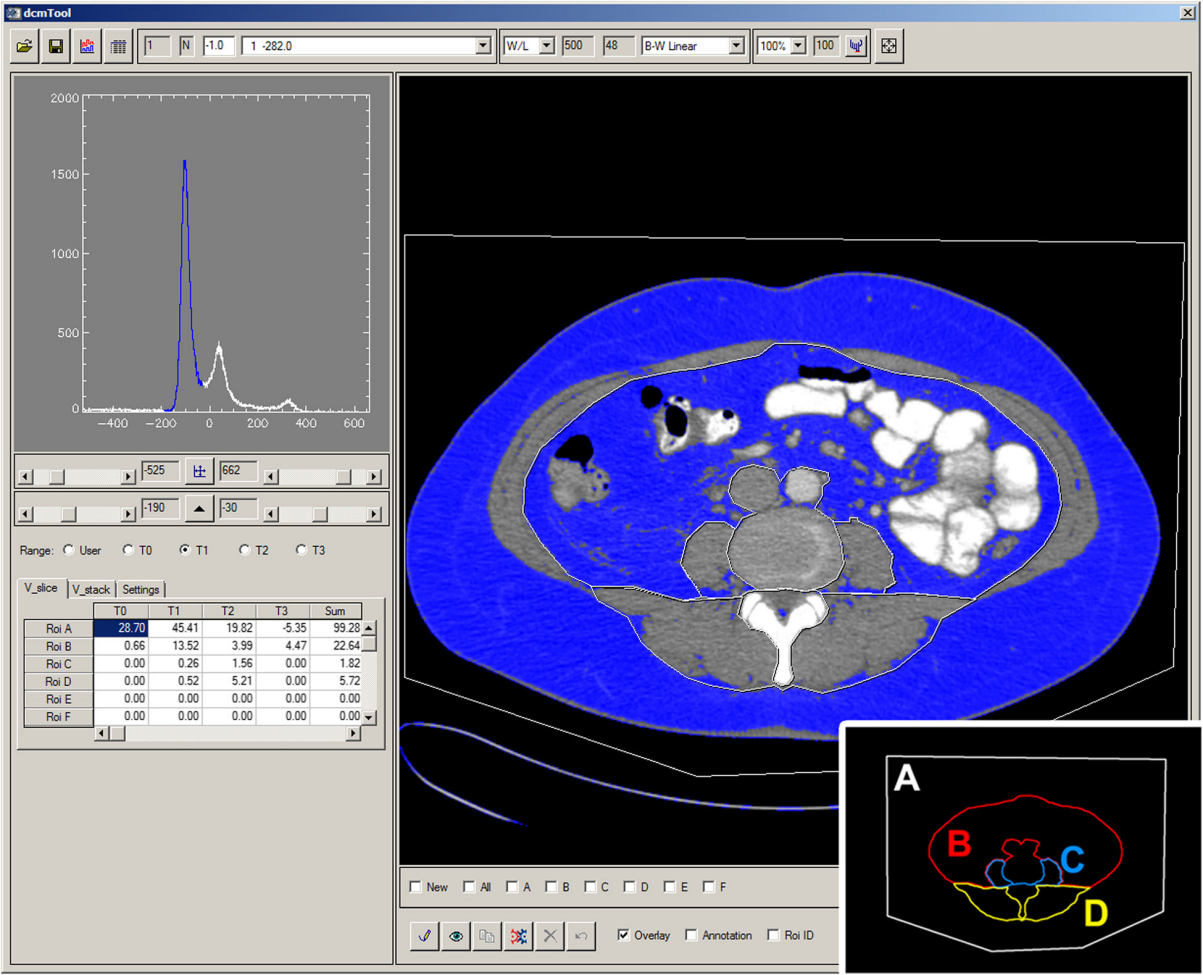
Software tool for quantification of Visceral and Subcutaneous Adipose Tissue areas. Axial CT slice at the level L3-L4 (right). ROIs are assigned as “A” for total abdominal tissue (A_TAT_), “B” for visceral adipose compartment (A_VAT_), “C” for M. psoas (A_MPSO_) and “D” for the paraspinal muscle compartments (A_MSPI_). Based on the histogram (left), voxel count can be adjusted by selection of lower and upper density limits, on this screenshot specific for adipose tissue (−190 to −30 Hounsfield Units); named tissue 1 (T1) and depicted as blue range in the histogram. The corresponding adipose tissue pixels are colored blue and resulting A_VAT_ is given in the table on the lower left. Note that intial values for the selected voxel volumes are corrected for slice thickness: e.g. for ROI A 45.63 cm^3^/ 1 mm slice thickness = 45.63 cm^3^/0.1 cm = 456.3 cm^2^

Segmentation was performed using a custom made DICOM quantification tool under IDL (Exelis, Boulder, CO, USA), which had shown high agreement with the commercially available software *sliceOmatic* 5.0 (Tomovision, Magog, Canada) in a yet unpublished internal validation. A screenshot of the applied segmentation software is shown in Fig. 2. Before segmentation slice thickness was standardized for each CT slice to fit 1 mm. Next a tissue specific range of density was defined in accordance with the literature. The specific density range for adipose tissue (AT) was set to − 190 to − 30 Hounsfield units [27]. Density based selection allows for the specific measurement of every AT-equivalent pixel within manual segmented ROIs, for total and visceral adipose tissue (TAT and VAT), resulting in cross sectional measurement in cm^2^. Other abdominal tissues and structure, such as bone, parenchymal organs or bowel are reliably excluded, having higher or lower density. For evaluation of skeletal muscle tissue a density range was set from − 30 to 150, as reported by others [27, 28].

Click-wise segmentation of regions of interest (ROIs) was performed manually. Results were exported in comma-separated value file format.

ROIs were defined in the following order (see Fig. 2). First the total body area was selected (ROI A), followed by the inner border of the abdominal wall, exclusive of the tissue surrounding aorta and inferior vena cava (ROI B). Third the psoas muscles were segmented and connected by overlapping, bridge-like lines at the anterior face of the spinal canal (ROI C). The last ROI was defined by the outer border of paravertebral muscles (ROI D), avoiding subcutaneous adipose tissue nearby. Adipose tissue in ROI A and B were defined as area of TAT (A_TAT_) and VAT (A_VAT_), respectively. Area of subcutaneous adipose (A_SAT_) was calculated as the difference of A_TAT_ and A_VAT_. Muscle tissue in ROI C and ROI D were set as area of Psoas muscle (A_MPSO_) and area of paraspinal muscle (A_MSPI_), respectively. Muscle tissue in ROI A excluding muscle tissue in all other ROIs yielded the area of the ventral abdominal wall muscle (A_MVEN_). These three muscle skeletal areas together summed up to the total muscle area (A_MTOT_).

The skeletal muscle index (SMI) was calculated as reported by Martin et al. [18]. Mean muscle attenuation was calculated as described by others [18] with the slight difference that only psoas and paraspinal muscle areas at the level L3-L4 were used, ignoring the ventral abdominal wall to avoid inexact muscle-fat differentiation in that region, e.g. due to ascites which usually has similar density values as muscle tissue. The image dependent definitions of sarcopenia were used as described in detail by Martin et al. [18].

### Statistical analysis

Data are presented as median (range) unless otherwise specified. Statistical differences between groups were determined by Mann-Whitney-U-Test (MWU). Binary logistic regression was performed for multivariate analysis of imaging parameters. Covariables were chosen based on the results of the univariate analysis and dependent (e.g. sarcopenia and MA) were restricted to one. Thereby the influence of sarcopenia (defined by MA or SMI), A_VAT_ and DPD on major complications or severe POPF was tested. Results are presented as odds ratios (OR) with standard error. All statistical analyses were performed with SPSS for Windows (version 20.0, SPSS Inc., Chicago, IL, USA). Figures were generated in Prism 7 (GraphPad Software, La Jolla, CA, USA) or Microsoft Excel (version 2010 Professional, Microsoft Corporation, Redmond, WA, USA). *P* values are reported with two decimals.

## Results

### Patient characteristics

A total of 347 pancreaticoduodenectomy operations were carried out at the local department of surgery. For 139 (40.1%) patients (55 females; 84 males) preoperative CT images were available and these patients were analyzed. Mean patient age and BMI were 60.4 (range 25.3–84.0) years and 25.4 (17.7–36.8) kg/m^2^, respectively.

The baseline characteristics and relevant categories of comorbidities are provided in Table 1. Gender and age showed no significant differences in both groups. BMI was higher in patients suffering major complications (*p* < 0.03) and POPF (*p* < 0.05). Preoperative cardiovascular and pulmonary comorbidities were associated by an increased risk of severe complications (*p* < 0.01 each). Pulmonary comorbidities also POPF grade B and C (p < 0.0). Additionally to these categories associations of severe complications could be observed for the diagnosis of preoperative metabolic syndrome (*p* < 0.09) and arterial hypertension (p0.01). Major POPF was linked to the diagnosis of arterial hypertension (0.01) and obesity (*p* < 0.06).

In regards to preoperative chemotherapy, there were only two cases with neoadjuvant treatment. Forty-four patients underwent adjuvant treatment. We were not able to obtain detailed data about the complete postoperative chemotherapy course. Note, most chemotherapies were performed by external oncologists in other institutions.treatment.

Most frequent surgical procedure was PPPD in 84 (60.4%) cases, followed by Kausch-Whipple in 55 (39.6%) cases. Thirty-three (23.7%) patients had an entirely uncomplicated postoperative outcome, 69 (49.6%) patients showed minor complications and in 37 (23.6%) cases major complications were seen.

## Imaging parameters

The majority of the CT (118 out of 139; 84.9%) was in portal venous contrast. Most relevant imaging data is also shown in Table 2. DPD and DPP could be measured in 125 (89.9%) and 136 (97.8%) patients, respectively. Median DPD was 2.9 mm (Range 0.7–10.7) and more narrow in patients with complications equal to or greater stadium IIIb (*p <* 0.04) and severe POPF (*<* 0.01). Median DPP value was 17.7 (6.9–37.9) mm and there was no significant difference regarding major complications or POPF.

**Table 2:** Univariate analysis of risk factors for complications and fistulas after pancreaticoduodenectomy

	Complication < IIIb	Complication IIIb ≤	*p<*	No Grade B or C fistula	Grade B or C fistula	*p<*
CT data						
A_VAT_ [cm^2^]	123	157	0.06	120	180.1	**0.01**
A_MPSO_ [cm^2^]	20.3	18.9	0.12	20.3	18.5	0.24
A_MSPI_ [cm^2^]	55.8	52.7	0.37	56.2	52.0	0.05
A_MVEN_ [cm^2^]	55.7	58.3	0.73	56.5	57.2	0.64
A_MTOT_ [cm^2^]	130	130.0	0.83	131	127	0.32
MA [HU]	40.7	33.0	**0.01**	40.3	33.1	**0.01**
SMI [cm^2^/m^2^]	44.5	44.9	0.77	44.9	43.4	0.30
DPP [mm]	17.3	19.3	0.14	17.5	18.7	0.55
DPD [mm]	3.00	2.40	**0.04**	3.00	2.00	**0.01**
**Sarcopenic**	9	28	**0.04**	4	22	**0.01**

Continuous parameters are reported as median, and categorical parameters are counted. DPP: Diameterof Pancreatic Parenchyma, DPD: Diameter of Pancreatic Duct; A_VAT_: Area of visceral adipose tissue; A_MPSO_: Area of psoas muscle; A_MSPI_: Area of dorsospinal muscles; A_MVEN_: Area of ventral abdominal muscles, A_MTOT_: total muscle area. *p*: level of significance was determined by the Mann-Whitney-U Test. bold numbers indicate *p* < 0.05

A_VAT_ could be quantified in all 139 patients with a median value of 127.5 (14.5–473.0) cm^2^. A_SAT_ could only be quantified in 124 (89.2%) patients and was not further evaluated. A_VAT_ was significantly larger in patients with severe POPF (*p* < 0.01), in regards to major complications only a trend was seen (*p* < 0.06).

Areas of psoas, paraspinal, ventral abdominal and total muscle (A_MPSO_, A_MSPI_, A_MVEN_ and A_MTOT_) showed no significant difference between major complications and POPF. Median muscle attenuation was both lower in groups with major complications (*p* < 0.01) and POPF (*p* < 0.01).

Median SMI was 44.7 (19.6–71.0) cm^2^/m^2^ in patients with or without major complications. There were no significant differences between the different groups mentioned above. Following the published thresholds for SMI and MA for prediction of survival [18] in our cohort there were 35 (11 females and 24 males) and 36 (14 females and 12 males) patients classified as sarcopenic, respectively, accounting for a total of 60 (21 females and 25 males) sarcopenic individuals. There were 28 sarcopenic out of 37 patients (75.7%) that developed major complications and 22 sarcopenic patients out of 26 patients (84.6%) that suffered from severe POPF.

Binary logistic regression was performed for prediction of major complications or severe POPF was tested. Included parameters were sarcopenia, A_VAT_ and DPD. For major complication no significant results were achieved. For severe POPF binary logistic regression revealed significant results for all three parameters sarcopenia (*p* < 0.03, OR 4.30, CI 1.154–16.005), A_VAT_ (*p* < 0.05, OR 1.006, CI 1.000–1.012) and DPD (*p* < 0.04, OR 0.725, CI 0.537–0.978).

**Fig. 3:**
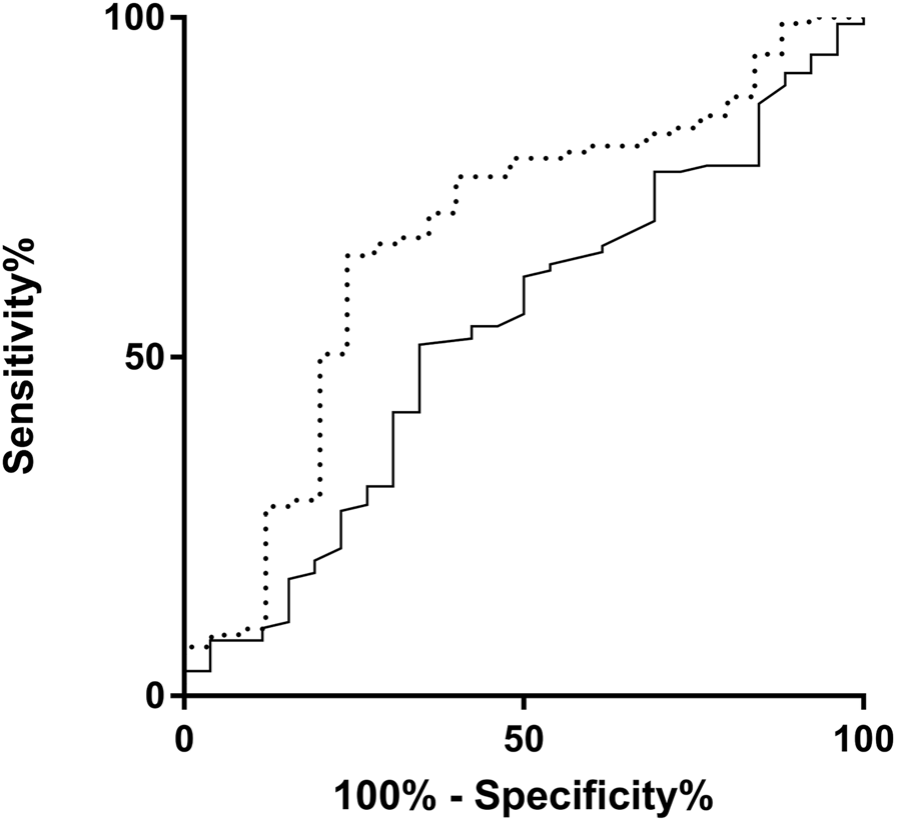
ROC analyses. Influence of mean attenuation (MA) and diameter of the pancreatic duct (DPD) on severe postoperative pancreatic fistula (POPF, grade B and C following the ISGPF classification). Dotted line indicates MA, continuous line indicates DPD. In the analysis of the Receiver operator characteristics (ROC) curve, the area under the curve (AUC) were 0.716 and 0.723 in regards to severe POPF, respectively

In the analysis of the Receiver operator characteristics (ROC) curve, the area under the curve (AUC) were 0.638 and 0.723 for DPD and MA, respectively, in regards to major complications (see Fig. 3). For severe POPF corresponding AUC were 0.716 and 0.723, respectively.

## Discussion

The morbidity rate after PD is still a relevant problem and many earlier reports tried to predict major complications perioperatively. In general there is still no standard approach to predict major postoperative complications and severe POPF. Several studies aimed to predict preoperatively POPF by using various CT parameters, such as VAT, pancreatic features and surrogate parameters reflecting sarcopenic obesity:

In our data we were first able to reproduce the known effect of visceral obesity on the development of complications and POPF as reported earlier by others [3–5, 7, 29, 30]. This is in line with the increasing number of publications that estimate VAT as an overall negative factor to human health by favoring cardiovascular complications known as the metabolic syndrome [31].

Next we demonstrated that the diameter of the pancreatic duct is negatively associated with complications and POPF after PD. This was reported earlier by various studies: Frozanpor used 2 mm [13], three independent publications by Rosso et al., Liu et al. and Su et al. used 3 mm [9, 15, 16], Fang et al. used 3.1 mm [17] and Callery et al. [11] as well as Sandini et al. [5] both used 5 mm as opimum cutoff values to predict POPF. To the contrary Schröder et al. [4] and Nishida et al. [8] did not see significant impact of DPD on POPF. In our data 69 out of 139 patients showed a duct diameter under 3 mm. 23 and 17 of them (33.3 and 24.6%) showed major complications or severe POPF, respectively. There were 14 (20%) and 9 (12.8%) cases of major complications or severe POPF in the 70 patients with a duct size over 3 mm. All in all this methods has some potential, but exactness of measurement is limited and specific cutoffs might therefore be hard to validate.

In contrast to existing studies, our analysis could not confirm that a pancreas thicker than 12 mm significantly increases the incidence of POPF, as shown after DP using stapler closure in 122 distal pancreatectomies [14]: In our cohort only 14 patients (8 women) had a DPP of under 12 mm. This comparison is limited by different surgical resection sites, though.

Several reports addressed the diameter or volume of the pancreatic parenchyma: Frozanpor et al. used data from 182 patients to show that a large pancreas volume is significantly higher in patients suffering from POPF [10]. In a similar approach Roberts et al. used data from 217 patients to show that the pancreas width is significantly higher in patients suffering from POPF [7]. This observation was validated in 266 patients by Nishida et al. [8]. In the data reported in this article, DPP was not relevant risk factor for complications. We did not assess pancreatic volume because this requires 3D software and therefore this method is too complex for a standard approach.

Nishida et al. also stated preoperative sarcopenia to favor POPF [8]: Authors included skeletal muscle areas and SMI into their analysis. Both were reduced in patients with POPF. In the same publication MA was not assessed and therefore characterization of sarcopenic obesity stayed incomplete. In our data MA was the strongest overall predictor of POPF. Therefore we can only speculate this discrepancy between our approach and the work Nishida et al. to be based on technical limitations since the most relevant publication on that issue came out in 2013 [18]: Martin et al. demonstrated that muscle depletion and low muscle attenuation can be used as surrogate parameters for cancer cachexia and thereby assessed for survival prognosis [18]. Additionally, Martin et al. provided threshold values for skeletal muscle index and mean attenuation to diagnose sarcopenia. Looking at our cohort we could only partially confirm these results: MA was associated the strongest with major complications and POPF. Following the thresholds for SMI and MA 4 (2 females and 2 males) and 56 (27 females, 29 males) patients were classified as sarcopen, respectively. Okumura et al. demonstrated psoas muscle mass index and intramuscular adipose tissue content to be associated with mortality after pancreatic resection in 230 patients [32]. Van Dijk et al. could link a low MA to reduction of survival in 199 patients [29]. Namm et al. showed similar results applying a semi-automated technique in 116 patients [33]. Sui et al. linked sarcopenia as defined by total muscle area to the 5 year survival [34]. Most recently Sugimoto et al. showed data from 323 patients and favored a sex-standardized skeletal muscle index as best factor of prediction of both overall and recurrence-free survival [35]. In another quite recent multi-center observation on 120 ptients, Pecorelli et al. stated sarcopenic obesity to be associated with failing rescue from major complications [36].

Sandini et al. in contrary contradicted these findings by normalizing the muscle area to height when they only found an index of both parameters to be predictive for complications [30]. Also contradicting most findings mentioned above, Clark et al. argued that more precise and robust measures of visceral fat, subcutaneous fat, and muscle mass fail to predict cancer progression. They see an explanation in the complexity of cancer biology [37].

In unresectable pancreatic cancers, Ishii et al. stated concluded that the psoas muscle index was the best predictor for survival [38]. Similar results were achieved by Rollins et al. [39]. Further work is needed to develop reliable and most likely gender-, age and ethnicity-dependent cutoff values.

Another question is how to quantify CT parameters in a most efficient way. For analysis of A_VAT_, skeletal muscle areas and muscle attenuation, the axial slice at the level of the intervertebral space L3-L4 was chosen. This selection refers to a variety of studies on large cohorts of normal weight to obese subjects, stating best agreement between VAT areas on single imaging slices and total VAT volume for levels cranial of L4-L5 intervertebral space [21, 40], more specific at level L3-L4. For simultaneous evaluation of skeletal muscle tissue and A_VAT_, also the level of L3 was shown to be most precise [26]. The software application used for segmentation in this project is easy to use and can be made available upon request. Overall segmentation time is under 5 min. Therefore the presented approach can smoothly be integrated in the clinical setting in order to evaluate sarcopenia on a large scale abdominal CT images.

The presented study has some limitations. First this was a retrospective descriptive study and results should be interpreted with care. Second patient number seems small: we used all consecutive CT datasets of a specific period of time available to perform this study. This resulted in 139 patients (55 women), which seems few to make general assumptions, but is in line with similar publications. In our study the main reason for this limitation was the lack of external CT scans. Due to legal issues, external CTs are not stored in the archive of our institution. Therefore we mainly relied of in-house imaging data.

Next, with a minority of 55 females of the 139 patients, there most likely is a potential gender bias. This can be explained by the also male-dominated prevalence of the of pancreatic adenocarcinoma, that is the most common disease that leads to PD.

Several comorbidities, especially a slightly increased BMI and arterial hypertension, indicate associations with postoperative complications and POPF which may introduce a selection bias. Besides patient-related limitations, there are technical drawbacks of our study: The segmentation of visceral and muscle tissue was accomplished using published density values. This ignores the actual variability in tissue density, e.g. due to ascites. Although this could have been addressed by individual histogram-dependent selection of the respective tissue, we restricted our analysis to the published density range to grant comparability with existing data. Another technical limitation is due to the often incomplete coverage of subcutaneous adipose tissue in the field of view of the CT scanner, especially in men with a large abdominal circumference. Therefore we had to exclude this parameter from our evaluation in order to avoid a selection bias. In future studies this might be overcome by using complementary methods for SAT quantification e.g. ultrasound [41, 42]. Furthermore, most datasets lacked native CT scans. These are mandatory for valid measurement of pancreatic density [3, 12]. With a majority of contrast-enhanced protocols in our CT data we could not quantify pancreatic density in an appropriate way, which is a general limitation in similar retrospective approaches. Last, in 14 cases DPD could not be quantified, most likely because of a very narrow duct, which biases the analysis towards extended DPD widths. The magnitude of this effect could be examined by setting the value of a non-viewable DPD to a standard width, e.g. the respective slice thickness.

Looking at other imaging techniques, magnetic resonance imaging and spectroscopy can replace histological assessment e.g. by Mutli-Echo-Dixon techniques [43]. Earlier it was showed that a fatty infiltration of is associated with POPF [9]. Taken together MR offers even more potential in predictinfg POPF than CT. The major drawback of MR is the generalization of the data, because it is often complex, technically heterogeneous, and above all rare in a common patient collective. Thus computed tomography stays the gold standard for image-based assessment before pancreatic surgery.

All in all and in comparison to existing studies, the presented approach is comprehensive and integrates the most reliable markers as the diameters of pancreatic parenchyma and ductus, areas of adipose and muscle tissue. The quantification of muscle attenuation is new in this field of interest.

## Conclusion

Besides the known factors visceral obesity and narrowness of the pancreatic duct, the mean muscle attenuation can easily be examined on routine preoperative CT scans and shows to predict POPF. Even though our study has some limitations, the data presented emphasizes that these parameters should be included in future prospective analyses of morbidity after pancreatic resection.

## Abbreviations

A_MPSO_: Area of M. psoas
A_MSPI_: Area of paraspinal muscles
A_MTOT_: Total area of muscle tissue
A_MVEN_: Area of ventral abominal muscles
ASA: American Society of Anaesthesiologists
A_SAT_: Area of subcutaneous adipose tissue
A_VAT_: Area of visceral adipose tissue
BMI: Body Mass Index (measured in kg/m^2^)
CT: Computed tomography
DPD: Diameter of the pancreatic duct
DPP: Diameter if the pancreatic parenchyma
ISGPF: International Study Group on Pancreatic Fistula Definition
MA: Mean muscle attenuation
POPF: Postoperative pancreatic fistula
PPPD: Pancreaticoduodenectomy
SAT: Subcutaneous adipose tissue
SMI: Skeletal muscle index
VAT: Visceral adipose tissue
